# Introducing co-review to *RSC Advances*

**DOI:** 10.1039/d4ra90099e

**Published:** 2024-10-01

**Authors:** Laura Fisher, Russell J. Cox, Karen Faulds

## Abstract

*RSC Advances* now offers the option of co-review for our reviewers. Editors-in-Chief Russell J. Cox and Karen Faulds, and Executive Editor Laura Fisher, offer more detail on how this will work.
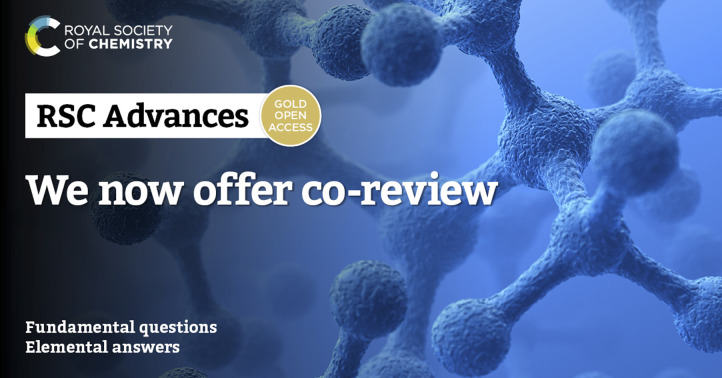

At *RSC Advances* we are committed to supporting researchers at all stages of their career, with a particular emphasis on supporting the future generation of researchers. As the RSC’s largest journal, we have always tried to be innovative and introduce ideas that enable opportunities for all. In 2017, we were the first RSC journal to convert to an APC-based open-access model.^1^ Our article-based publication process gives authors fast and immediate page numbers, our topic-modelling system characterises our papers by subject area so that readers can find what they are looking for in such a broad chemistry journal, and we pioneered the use of Proof Central to simplify the proofreading process.^2^ Our Reviewer Panel enables us to quickly find reliable reviewers, as well as giving the opportunity for early-career and under-represented groups to review,^3^ and our recently launched Student Paper Awards recognise students who have made a significant contribution to published work.^4^

In 2023, we were also delighted to introduce the option of transparent peer review for our authors – where anonymous reviewers’ comments, editor’s decision letters, and the authors’ own responses to these are published alongside the main article.^5^ Authors are free to opt in or out of transparent peer review at any stage during the peer review process, up to acceptance, and reviewers will be informed that their anonymous comments will be published alongside the article should the authors choose to opt-in.

In line with our aim of introducing innovative ideas into the journal, we are now excited to offer the option of co-review at *RSC Advances*. Co-review allows two individuals to collaborate on the peer review of a manuscript, with both reviewers eligible for recognition through the Web of Science Reviewer Recognition Service.

Usually, when an early-career researcher writes a reviewer report on behalf of or in collaboration with their supervisor, it is usually only the supervisor, as the individual invited to review, who receives credit for the report. The introduction of co-review on *RSC Advances* provides a way for both parties to receive credit for their contribution.

Co-review enables early-career researchers to develop their skills in peer review through collaboration with an experienced colleague and to demonstrate experience that may further their career. Please note that co-reviewers must meet the RSC’s peer reviewer eligibility criteria in order to review for *RSC Advances*. More information can be found at our Author and Reviewer Hub.^6^

You can read further information about the peer review options on our website.^7^

“We are really excited to launch this new initiative and to give early-career researchers the opportunity to take part in, and importantly, get recognition for their contribution to peer review. *RSC Advances* strives to ensure excellent peer review for all our authors and this new initiative, alongside transparent peer review, gives a strong opportunity to train and support the next generation of reviewers” – Karen Faulds, Editor-in-Chief, *RSC Advances*

“The *RSC Advances* Reviewer Panel is at the heart of our journal. We now want to propagate that reviewing experience to the next generation of scientists, and co-review will be a great way to do this. Innovations in ScholarOne will allow experienced reviewers to pass on their expertise to more junior colleagues, and for both to get credit for the work” – Russell J. Cox, Editor-in-Chief, *RSC Advances*

Karen Faulds, Editor-in-Chief, University of Strathclyde, UK

Russell J. Cox, Editor-in-Chief, Leibniz Universität Hannover, Germany

Laura C. Fisher, Executive Editor, *RSC Advances*
